# Self-correction of posture: assessment of the quality of the movement accomplished by non-instructed school children

**DOI:** 10.1186/1748-7161-7-S1-O66

**Published:** 2012-01-27

**Authors:** L Stolinski, T Kotwicki

**Affiliations:** 1 Rehasport Clinic, Poznan, Poland; Sports Secondary School Complex the John Paul II, Skierniewice, Poland; 2 Spine Disorders Unit Department of Pediatric Orthopedics and Traumatology, University of Medical Sciences, Poznan, Poland; 3 Sports Secondary School Complex the John Paul II, Skierniewice, Poland

## Purpose of the study

To assess how the movement of self-correction of the posture is accomplished by non-instructed school children.

## Background

Postural defects are common in school children and expose them to repetitive claims from the adults to actively correct the posture. Usually the command to “straighten the back” is expressed.

## Material and methods

126 primary school pupils, 60 girls and 66 boys, aged 7.0 to 13.0 years (9.1±1.6), were examined in standing position twice: in a relaxed posture and in actively corrected posture (after the “straight the back” command). Children were not instructed what corrected posture means. Spinous processes of C7, Th6, Th12 and S1 were clinically identified. Sagittal clinical angles: C7-Th6 (upper thoracic kyphosis, UTK), Th6-Th12 (lower thoracic kyphosis, LTK), Th12-S1 (lumbar lordosis, LL) and sacral inclination (SI) were measured with Rippstein plurimeter [1-4]. Significance of difference of the means was checked with paired t-test.

## Results

The UTK, LTK, LL and SI angles in relaxed versus corrected posture were as follows: 32.4°±5.3° versus 29.3°±6.8° (difference significant), 6.5°±7.8° versus -2.3°±8.1° (difference extremely significant), 34.8°±7.9° versus 33.6°±8.3° (not significant) and 23.5°±5.9° versus 25.8°±5.5° (difference significant), respectively. Girls and boys followed the similar pattern of changes.

## Conclusion

Non-instructed school children straighten their back by introducing pathological lordosis in the lower thoracic spine. Instead, they do pelvic anteversion and only slightly correct upper thoracic kyphosis. Adults’ commands correcting posture may be not beneficial for the children.
